# Survey of Adherence with COVID-19 Prevention Behaviors During the 2020 Thanksgiving and Winter Holidays Among Members of the COVID-19 Community Research Partnership

**DOI:** 10.1007/s10900-021-01021-z

**Published:** 2021-08-12

**Authors:** James E. Peacock, David M. Herrington, Sharon L. Edelstein, Austin L. Seals, Ian D. Plumb, Sharon Saydah, William H. Lagarde, Michael S. Runyon, Patrick D. Maguire, Adolfo Correa, William S. Weintraub, Thomas F. Wierzba, John W. Sanders, for The COVID-19 Community Research Partnership Study Group, John Walton, Mark A. Espeland, Morgana Mongraw-Chaffin, Alain Bertoni, Martha A. Alexander-Miller, Allison Mathews, Iqra Munawar, Brian Ostasiewski, Christine Ann Pittman Ballard, Metin Gurcan, Alexander Ivanov, Allison Matthews, Giselle Melendez Zapata, Marlena Westcott, Karen Blinson, Laura Blinson, Douglas McGlasson, Mark Mistysyn, Donna Davis, Lynda Doomy, Perrin Henderson, Alicia Jessup, Kimberly Lane, Beverly Levine, Jessica McCanless, Sharon McDaniel, Kathryn Melius, Christine O’Neill, Angelina Pack, Ritu Rathee, Scott Rushing, Jennifer Sheets, Sandra Soots, Michele Wall, Samantha Wheeler, John White, Lisa Wilkerson, Rebekah Wilson, Kenneth Wilson, Deb Burcombe, Lewis H. McCurdy, Michael A. Gibbs, Yhenneko J. Taylor, Lydia Calamari, Hazel Tapp, Amina Ahmed, Michael Brennan, Lindsay Munn, Keerti L. Dantuluri, Timothy Hetherington, Lauren C. Lu, Connell Dunn, Melanie Hogg, Andrea Price, Mariana Leonidas, Laura Staton, Kennisha Spencer, Melinda Manning, Whitney Rossman, Frank Gohs, Anna Harris, Bella Gutnik, Jennifer S. Priem, Ryan Burns, Kristen Miller, Chris Washington, Allison Moses, Sarahfaye Dolman, Julissa Zelaya-Portillo, John Erkus, Joseph Blumenthal, Ronald E. Romero Barrientos, Sonita Bennett, Shrenik Shah, Shrey Mathur, Christian Boxley, Paul Kolm, La Long, Cheng Zhang, Eva Hochberger, Ella Franklin, Deliya Wesley, Naheed Ahmed, Richard Oberhelman, Joseph Keating, Patricia Kissinger, John Schieffelin, Joshua Yukich, Andrew J. Beron, Devin Hayes, Johanna Teigen, Karen Kotloff, Wilbur Chen, DeAnna Friedman-Klabanoff, Andrea Berry, Helen Powell, Lynnee Roane, Reva Datar, Leandro Mena, Bhagyashri Navalkele, Yuan I. Min, Alexandra Castillo, Lori Ward, Robert P. Santos, Courtney Gomillia, Pramod Anugu, Yan Gao, Jason Green, Ramona Sandlin, Donald Moore, Lemichal Drake, Dorothy Horton, LaMonica Daniel, Charin L. Hanlon, Lynette McFayden, Isaura Rigo, Kelli Hines, Lindsay Smith, Alexa Drilling, Monique Harris, Belinda Lissor, Vivian Cook, Maddy Eversole, Terry Herrin, Dennis Murphy, Lauren Kinney, Polly Diehl, Nicholas Abromitis, Tina St. Pierre, Judy Kennedy, Lauren Kinney, Bill Heckman, Denise Evans, Vivian Cook, Maddy Eversole, Julian March, Ben Whitlock, Wendy Moore, Shakira Henderson, Thomas R. Gallaher, Michael Zimmer, Danielle Oliver, Tina Dixon, Kasheta Jackson, Martha Reavis, Monica Menon, Brandon Bishop, Rachel Roeth, Mathew Johanson, Alesia Ceaser, Amada Fernandez, Carmen Williams, Jeremiah Hargett, Keeaira Boyd, Kevonna Forbes, Latasha Thomas, Markee Jenkins, Monica Coward, Derrick Clark, Omeshia Frost, Angela Darden, Lakeya Askew, Sarah Phipps, Victoria Barnes, Robin King-Thiele, Terri S. Hamrick, Abdalla Ihmeidan, Chika Okafor, Regina B. Bray Brown, Poornima Vinod, Lawrence Klima, Amber Brewster, Danius Bouyi, Katrina Lamont, Kazumi Yoshinaga, A. Suman Peela, Giera Denbel, Jason Lo, Mariam Mayet-Khan, Akash Mittal, Reena Motwani, Mohamed Raafat, Evan Schultz, Aderson Joseph, Aalok Parkeh, Dhara Patel, Babar Afridi, Diane Uschner, Michele Santacatterina, Greg Strylewicz, Brian Burke, Mihili Gunaratne, Meghan Turney, Qin Zhou, Rebecca Laborde, Anne McKeague, Grace Tran, Johnathan Ward, Joyce Dieterly, Nana Darko, Kimberly Castellon, Isabella Malcolm, Ryan Brink, Atira Goodwin, Ruth Berkelman, Kimberly Hanson, Scott Zeger, Johns Hopkins, Cavan Reilly, Kathy Edwards, Helene Gayle

**Affiliations:** 1 Department of Internal Medicine, Section on Infectious Diseases, Medical Center Blvd, Wake Forest School of Medicine, Winston-Salem, NC 27157 USA; 2 Department of Internal Medicine, Section on Cardiovascular Medicine, Wake Forest School of Medicine, Winston-Salem, NC USA; 3 Biostatistics Center, George Washington University Milken School of Public Health, Washington, DC USA; 4 Centers for Disease Control and Prevention, Atlanta, GA USA; 5 Wake Med Health and Hospitals, Raleigh, NC USA; 6 Atrium Health, Charlotte, NC USA; 7 New Hanover Regional Medical Center, Wilmington, NC USA; 8 University of Mississippi School of Medicine, Jackson, MS USA; 9 MedStar Health Research Institute and Georgetown University, Washington, DC USA

**Keywords:** COVID-19, Holiday travel, Gatherings, Prevention behaviors

## Abstract

Prevention behaviors represent important public health tools to limit spread of SARS-CoV-2. Adherence with recommended public health prevention behaviors among 20000 + members of a COVID-19 syndromic surveillance cohort from the mid-Atlantic and southeastern United States was assessed via electronic survey following the 2020 Thanksgiving and winter holiday (WH) seasons. Respondents were predominantly non-Hispanic Whites (90%), female (60%), and ≥ 50 years old (59%). Non-household members (NHM) were present at 47.1% of Thanksgiving gatherings and 69.3% of WH gatherings. Women were more likely than men to gather with NHM (p < 0.0001). Attending gatherings with NHM decreased with older age (Thanksgiving: 60.0% of participants aged < 30 years to 36.3% aged ≥ 70 years [p-trend < 0.0001]; WH: 81.6% of those < 30 years to 61.0% of those ≥ 70 years [p-trend < 0.0001]). Non-Hispanic Whites were more likely to gather with NHM than were Hispanics or non-Hispanic Blacks (p < 0.0001). Mask wearing, reported by 37.3% at Thanksgiving and 41.9% during the WH, was more common among older participants, non-Hispanic Blacks, and Hispanics when gatherings included NHM. In this survey, most people did not fully adhere to recommended public health safety behaviors when attending holiday gatherings. It remains unknown to what extent failure to observe these recommendations may have contributed to the COVID-19 surges observed following Thanksgiving and the winter holidays in the United States.

## Introduction

After declining following a summer surge, infections due to severe acute respiratory syndrome coronavirus-2 (SARS-CoV-2) precipitously increased across the United States with over 1.87 million cases reported during the month of October 2020 [1]. New cases exceeded 100000 per day for the first time on 30 October [2]. With Thanksgiving approaching, it was widely reported in the news media that Thanksgiving gatherings might serve as superspreader events, further exacerbating the surge in coronavirus disease-19 (COVID-19) cases [3]. Guidelines for Thanksgiving travel and safe holiday gatherings were issued by the Centers for Disease Control and Prevention (CDC) on 12 November [4]. Guidance included recommendations to minimize travel, to take steps to prevent transmission if gathering with non-household members (NHM), and to limit the number of guests at gatherings. At any type of gathering, all attendees were encouraged to wear masks, observe social distancing guidelines, wash their hands often, and gather outdoors if possible [4]. Despite recommendations designed to decrease the risk of SARS-CoV-2 transmission by attending celebrations only with people in the household and deferring travel, the Associated Press reported that 1.17 million travelers passed through US airports on the Sunday after Thanksgiving [5]. Predictions of a post-Thanksgiving surge proved accurate [6, 7], so the CDC again cautioned Americans about travel and gatherings over the winter holiday (WH) season [8] and encouraged a broad range of safety precautions. As emphasized by the media, the potential consequences of WH travel and gatherings might be “catastrophic” [9], a concern that again proved prescient with COVID-19 cases peaking during the second week of January [10].

To assess participation in gatherings over Thanksgiving and during the WH season, especially those involving NHM, and to collect information about the use of measures to prevent exposure to, and transmission of, SARS-CoV-2 during gatherings, we surveyed > 20,000 participants in the COVID-19 Community Research Partnership (CCRP) after Thanksgiving 2020 and again in early January 2021. The results of those two surveys form the basis for this report.

## Methods

**Fig. 1:**
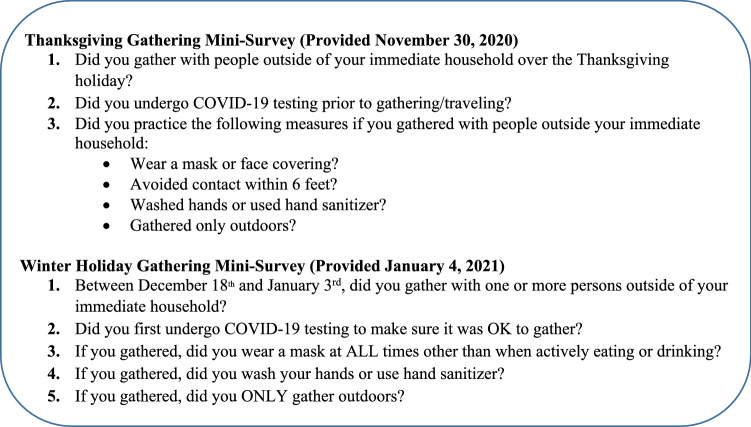
Questions included in electronic surveys completed by study participants following Thanksgiving and the winter holidays

The CCRP is a COVID-19 syndromic surveillance program approved by the Wake Forest School of Medicine Institutional Review Board. This activity was also reviewed by CDC and was conducted consistent with applicable federal law and CDC policy. Participants were recruited through direct email outreach to enroll patient populations from health care systems at six sites (Table 1): Wake Forest Baptist Health in the Winston-Salem NC, USA area, Atrium Health in the Charlotte NC, USA area, WakeMed in the Raleigh NC, USA area, MedStar Health in the Washington DC, USA area, New Hanover Regional Medical Center in the Wilmington NC, USA area, and a small number of participants at the University of Mississippi in the Oxford MS, USA area. Participants provided informed consent through an online consenting system. Over 32000 adults 18 years and older had volunteered to participate in the partnership by 24 December, 2020 with most originating from the mid-Atlantic (Washington DC area) and southeastern (North Carolina) United States. Beginning in April, 2020, CCRP participants began receiving a brief 5-question daily electronic survey via text or e-mail asking the recipient to report their health status, including questions about COVID-19 symptoms, testing, and diagnoses. To assess attendance at Thanksgiving and WH gatherings with NHM and use of recommended safety behaviors to prevent COVID-19, supplemental mini-surveys consisting of questions focused upon holiday behaviors were added to the standard daily survey on 30 November, 2020 covering the 4-day Thanksgiving holiday and on 4 January, 2021 covering the 2-week winter holiday period (Fig. 1). It should be noted that the opportunity for COVID-19 vaccination was limited at the time of the two surveys.

**Table 1:** Percentage of people in selected regions of the southeastern United States who gathered with persons outside their immediate household at Thanksgiving, 2020, and during the winter holiday, 2020–2021, according to age, sex, race/ethnicity, healthcare worker status, and study site

	Did you gather with people outside your immediate household over Thanksgiving?							Did you gather with people outside your immediate household over the winter holiday?						
	Total	No		Yes		RR (95%CI)	p-value	Total	No		Yes		RR (95% CI)	p-value
	n	n	Percent	n	Percent			n	n	Percent	n	Percent		
Total	20281	10721	52.9	9560	47.1			26841	8249	30.7	18592	69.3		
Sex														
Women	13985	7204	51.5	6781	48.5	1.10 (1.06, 1.14)	< 0.0001	18579	5507	29.6	13072	70.4	1.05 (1.03, 1.07)	< 0.0001
Men	6296	3517	55.9	2779	44.1	Ref		8262	2742	33.2	5520	66.8	Ref	
Age group (years)														
< 30	1151	460	40.0	691	60.0	1.65 (1.55, 1.77)	< 0.0001	1375	253	18.4	1122	81.6	1.34 (1.29, 1.39)	< 0.0001
30–39	3253	1402	43.1	1851	56.9	1.57 (1.48, 1.66)		4313	956	22.2	3357	77.8	1.28 (1.24, 1.32)	
40–49	3926	2020	51.5	1906	48.5	1.34 (1.26, 1.42)		5351	1568	29.3	3783	70.7	1.16 (1.12, 1.20)	
50–59	4405	2357	53.5	2048	46.5	1.28 (1.21, 1.36)		5640	1833	32.5	3807	67.5	1.11 (1.07, 1.14)	
60–69	4618	2616	56.6	2002	43.4	1.20 (1.13, 1.27)		6282	2124	33.8	4158	66.2	1.09 (1.05, 1.12)	
≥ 70	2928	1866	63.7	1062	36.3	Ref		3880	1515	39.0	2365	61.0	Ref	
Race/ethnicity														
White (not Hispanic/Latino)	18235	9427	51.7	8808	48.3	1.54 (1.40, 1.70)	< 0.0001	24072	7129	29.6	16943	70.4	1.28 (1.22, 1.35)	< 0.0001
Hispanic or Latino	455	265	58.2	190	41.8	1.34 (1.15, 1.55)		604	224	37.1	380	62.9	1.15 (1.06, 1.24)	
Other	720	431	59.9	289	40.1	1.28 (1.12, 1.46)		1003	371	37.0	632	63.0	1.15 (1.07, 1.23)	
Black or African American	871	598	68.7	273	31.3	RAef		1162	525	45.2	637	54.8	Ref	
Study site														
WakeMed	565	249	44.1	316	55.9	1.40 (1.22, 1.61)	< 0.0001	2524	708	28.1	1816	71.9	1.09 (1.05, 1.13)	< 0.0001
New Hanover	276	123	44.6	153	55.4	1.39 (1.27, 1.53)		653	161	24.7	492	75.3	1.14 (1.08, 1.20)	
Atrium Health	5260	2639	50.2	2621	49.8	1.25 (1.14, 1.37)		5477	1695	30.9	3782	69.1	1.04 (1.01, 1.08)	
Wake Forest Baptist Health	13462	7278	54.1	6184	45.9	1.15 (1.02, 1.31)		15729	4869	31.0	10860	69.0	1.04 (1.01, 1.07)	
U Mississippi	0							87	17	19.5	70	80.5	1.21 (1.09, 1.35)	
MedStar Health Research Institute	718	432	60.2	286	39.8	Ref		2371	799	33.7	1572	66.3	Ref	
Healthcare worker														
Yes	6002	2959	49.3	3043	50.7	1.11 (1.08, 1.15)	< 0.0001	7464	2210	29.6	5254	70.4	1.02 (1, 1.04)	< 0.0001
No	14279	7762	54.4	6517	45.6	Ref		19377	6039	31.2	13338	68.8	Ref	

Chi-square tests were used to compare frequency of gathering and safe behaviors across demographic groups, and Mantel–Haenszel Chi-square test for trend was used across age groups

RR = risk ratio; risk ratios compared percentages in each category to the reference category indicated with 95% confidence intervals computed based on the asymptotic normality of maximum likelihood estimators

### Statistical Methods

Chi-square tests were used to compare completion of supplemental questionnaires, frequency of gathering, and safe behaviors among those who gathered, across demographic groups, with Mantel–Haenszel Chi-square used across age groups; risk ratios (95% confidence intervals) compared percentages in each category to the reference category indicated. Multivariable logistic regression models were used to identify variables that independently predict gathering with NHM at each of the holidays, and among those who gathered to independently predict mask wearing during the gatherings, adjusting for age, sex, race/ethnicity, study site and healthcare worker status in both models. For Thanksgiving only, participants who reported three or more specified behaviors (pre-gathering COVID-19 testing, mask wearing, social distancing, hand washing, and outdoor gatherings) were compared to those who reported none or one behavior. Confidence limits for the estimated parameters were computed based on the asymptotic normality of maximum likelihood estimators. SAS, Version 9.4 (SAS Institute, Cary, NC, USA) was used for all analysis.

## Results

Of 25427 participants enrolled in the CCRP by 30 November, 2020, 20281 (79.8%) completed the post-Thanksgiving mini-survey, 26841 of 32031 participants (83.8%) enrolled by 24 December, 2020 completed the post-WH survey, and 19354 participants responded to both surveys. The majority of respondents were non-Hispanic White (Thanksgiving: 89.7%, WH: 89.0%), female (Thanksgiving: 70.2%; WH: 70.0%), and aged ≥ 50 years (Thanksgiving: 52.2%; WH: 52.5%). Healthcare workers comprised 30.8 and 29.0% of the study population at Thanksgiving and WH, respectively. Response rates differed by sex (Thanksgiving: 80.3% men vs. 75.6% women; WH: 83.0% men vs. 80.3% women; both p < 0.0001), age (Thanksgiving: decreasing from 90.2% in those age ≥ 70 years to 57.4% in those < 30 years; WH: decreasing from 90.8% in those age ≥ 70 years to 62.6% in those < 30 years; both p-trend < 0.0001), and healthcare worker status (Thanksgiving: 78.3% non-healthcare worker vs. 74.0% healthcare worker; WH: 82.2% non-healthcare worker vs. 78.3% healthcare workers; both p < 0.0001). A total of 9560 (47.1%) respondents indicated that they attended gatherings with NHM during Thanksgiving, whereas a higher percentage of respondents (69.3%) attended gatherings with NHM over the WH.

Table 1 compares demographic characteristics of those participating and not participating in gatherings with NHM over Thanksgiving and during the WH. Women were slightly more likely to gather with persons outside their household than were men (Thanksgiving: 48.5 vs. 44.1%, p < 0.0001; WH: 70.4 vs. 66.8%, p < 0.0001). The percent of participants who attended gatherings with NHM decreased with increasing age (Thanksgiving: 60.0% of participants aged < 30 years to 36.3% of participants aged ≥ 70 years [p-trend < 0.0001]; WH: 81.6% of participants aged < 30 years to 61% of participants aged ≥ 70 years [p-trend < 0.0001]). Gathering with NHM differed across race/ethnic group (p < 0.0001) with non-Hispanic White participants more likely to gather with NHM than were Hispanic or Latino, or non-Hispanic Black or African American participants during both Thanksgiving and the WH (Thanksgiving: 48.3 vs. 41.8% and 31.3%, respectively; WH: 70.4 vs. 62.9% and 54.8%, respectively). Healthcare workers more frequently participated in gatherings with NHM than did non-healthcare workers (Thanksgiving: 50.7 vs. 45.6%, p < 0.0001; WH: 70.4% vs. 68.8, p < 0.0001). At both holidays, participants from MedStar Health Research Institute, which encompasses the Washington, DC, USA metro area, were least likely to hold gatherings with members outside their household (p < 0.0001). During both holidays, women, older age, non-Hispanic Black or African American race, Hispanic or Latino ethnicity, and enrollment from MedStar Health and, during the WH, occupation as a healthcare worker, were independently predictive of not gathering with NHM (data not shown).

**Fig. 2:**
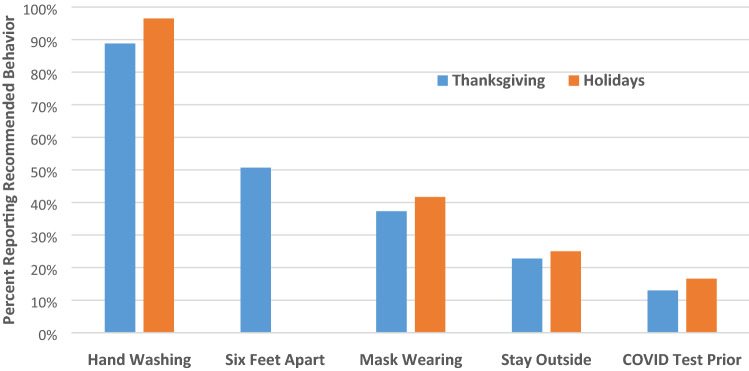
Frequency of respondents who reported observing specific recommended safeguards to prevent COVID-19 among respondents who reported gathering with people outside their immediate household over Thanksgiving and the winter holidays. The six feet apart question was not asked in the winter holiday survey

Prevalence of specified behaviors (pre-gathering COVID testing, frequent hand hygiene, social distancing, masking, gathering outdoors) among those who gathered with NHM during both Thanksgiving and the WH is summarized in Fig. 2. Of those gathering with NHM, 13.0 and 16.7% underwent testing for COVID-19 prior to gathering at Thanksgiving and the WH, respectively. Among those gathering during the Thanksgiving holiday, 88.8% reported washing hands or using hand sanitizer, 50.7% avoided contact with others within 6 feet, and 37.3% indicated that they wore a mask or face covering. Twenty-three percent of participants who reported gatherings with NHM indicated that gatherings were held outdoors. During the WH, 96.7% reported washing hands or using hand sanitizer and 41.9% wore face masks; gathering outdoors only was reported for 25.1% of gatherings. At Thanksgiving, three or more specified behaviors were reported by 38% of respondents whereas 37% utilized only one or none. During both holidays, age ≥ 70 years, non-Hispanic Black or African American race, Hispanic or Latino ethnicity, enrollment from MedStar Health (compared with Wake Forest and Atrium), and occupation as a healthcare worker were independently associated with wearing a mask or face covering, among those gathering with NHM (Table 2).

**Table 2:** Logistic regression models predicting mask wearing during gatherings, among participants who gathered with non-household members at Thanksgiving, 2020 (n = 9560) and over the winter holiday, 2020–2021 (n = 18592)

Variable	Thanksgiving		Winter holiday	
	Odds ratio (95% CI)	p-value	Odds ratio (95% CI)	p-value
Sex (men vs. women)	1.13 (1.02, 1.24)	0.2503	1.12 (1.01, 1.23)	0.0258
Age group				
Age < 30 vs. ≥ 70 years	0.38 (0.3, 0.47)	< 0.0001	0.36 (0.28, 0.45)	< 0.0001
Age 30–39 vs. ≥ 70 years	0.42 (0.35, 0.49)	< 0.0001	0.41 (0.35, 0.49)	< 0.0001
Age 40–49 vs. ≥ 70 years	0.49 (0.41, 0.57)	< 0.0001	0.49 (0.41, 0.58)	< 0.0001
Age 50–59 vs. ≥ 70 years	0.64 (0.55, 0.75)	< 0.0001	0.63 (0.54, 0.74)	< 0.0001
Age 60–69 vs. ≥ 70 years	0.73 (0.62, 0.85)	0.0028	0.72 (0.61, 0.84)	< 0.0001
Study site				
Atrium vs. MedStar	0.61 (0.47, 0.78)	< 0.0001	0.6 (0.46, 0.77)	< 0.0001
New Hanover vs. MedStar	0.76 (0.51, 1.15)	0.1466	0.76 (0.5, 1.15)	0.1911
Wake Forest vs. MedStar	0.68 (0.53, 0.87)	< 0.0001	0.66 (0.52, 0.85)	0.0013
WakeMed vs. MedStar	0.78 (0.56, 1.10)	0.0504	0.7 0(0.49, 1.00)	0.0520
Race/ethnicity				
Non-Hispanic black or African American vs non-Hispanic white	2.71 (2.11, 3.48)	< 0.0001	2.54 (1.96, 3.28)	< 0.0001
Hispanic vs non-Hispanic White	1.45 (1.08, 1.96)	0.1604	1.43 (1.06, 1.93)	0.0208
Other vs non-Hispanic white	1.38 (1.09, 1.76)	0.2359	1.36 (1.06, 1.75)	0.0161
Healthcare worker (Y vs. N)	1.31 (1.18, 1.44)	< 0.0001	1.31 (1.19, 1.45)	< 0.0001
Pre-gathering COVID-19 test (Y vs. N)	1.23 (1.09, 1.39)	0.1449	1.25 (1.1, 1.42)	0.0006

Based on separate logistic regression models at Thanksgiving and the winter holidays, adjusted for all variables listed

## Discussion

This survey provides a unique snapshot of behaviors associated with the Thanksgiving and WH seasons among persons in the eastern US. A large percentage of respondents (47.1%) reported gathering with members outside their immediate household over Thanksgiving and an even larger percentage (69.3%) did so over the longer WH. Among those who gathered with NHM, adherence to COVID-19 transmission prevention strategies other than handwashing or using hand sanitizer, were infrequent. Notably, the majority of people who gathered did not wear masks, one of the most important prevention strategies [11]. It is recognized that continually wearing masks is difficult since many holiday activities are centered upon eating which requires removal of masks. Even given the reality of the necessity to frequently remove masks for eating, our data raise concerns that consistent use of masks may not have occurred at times other than meals. In general, older participants, participants of non-Hispanic Black or African American race, Hispanic or Latino ethnicity, and those living in the urban DC area were less likely to gather with NHM and more likely to adhere to mask wearing during these gatherings than younger and non-Hispanic White participants and those from other sites. This observation suggests that holiday behaviors were modified in those groups in recognition of the fact that older age, racial minority groups, and Hispanic or Latino ethnicity are major risk factors for severe COVID-19 infection [2]. In contrast, respondents aged < 30 years old were most likely to attend gatherings with NHM, consistent with the hypothesis that many younger persons consider themselves to be at low risk for infection, especially serious infection [12]. Following Thanksgiving travel and gatherings [7], an ongoing increase in COVID-19 cases occurred across the US and that surge accelerated after the WH season [10]. Although a causal relationship was not established, lack of compliance with recommended prevention guidance, as was demonstrated by our survey, may have been a factor in this increase.

Several of our observations are consistent with a similar survey from Johns Hopkins which targeted Thanksgiving travel only [13]. In this survey of 7905 individuals from ten US states, 25.9% of respondents spent Thanksgiving outside their own home and 27.3% celebrated Thanksgiving with at least one NHM. Of note, mask wearing was practiced by nearly two-thirds of those who gathered with NHM or who traveled outside the home for Thanksgiving.

Maintaining high levels of adherence with public health guidelines and instructions is often challenging and is dependent upon multiple sociodemographic variables [14]. A recently published research letter examined changes in reported adherence with non-pharmaceutical interventions (NPI) for mitigation of COVID-19 in the US [15]. Using national surveillance data from the Coronavirus Tracking Survey for the period of April to November of 2020, those authors noted that there was a significant decrease in the NPI adherence index from 70 to 60% during the study period which ended in late November (i.e., around Thanksgiving) [15]. Protective behaviors for which adherence decreased over time included remaining at home except for essential activities, avoiding contact with members outside their household, and not having visitors in the home, all of which were “risk behaviors” assessed by the CCRP study cohort during the Thanksgiving and WH seasons.

An increased proportion of our survey participants chose to gather with members outside their immediate household over the WH season as compared to Thanksgiving. Possibilities for this observation include the longer WH season allowing more opportunity to hold gatherings with NHM, “pandemic fatigue” [16] including a general decline with observance of distancing and interaction guidelines over time [15, 17], and the desire of many Americans to participate in traditional holiday gatherings even in the face of strong recommendations not to do so [2, 4, 8]. It remains unclear as to what extent COVID-19 pandemic fatigue, coupled with increasing proportions of the population being vaccinated, will influence behaviors over the summer 2021 holidays. However, it is important to recognize that incomplete attainment of population immunity due to lack of vaccine confidence [18], combined with the emergence of more transmissible and potentially vaccine-resistant variants [19, 20], means that adherence to protective behaviors may still be important for some time to come. That message may require further refinement since guidelines about travel, gatherings, and “safeguard behaviors” were repeatedly emphasized prior to the Thanksgiving and WH [2, 4, 8], yet many persons in our study cohort failed to observe those guidelines and behaviors.

Our surveys had several limitations. Participants in the CCRP are restricted geographically to the mid-Atlantic and southeastern US. Behaviors of that population of participants may differ from those living in other areas of the US or the world. The survey is based on self-reported data which cannot be independently verified for accuracy. Participation in the CCRP and the mini-survey completion were voluntary, and the mini-surveys were more likely to be completed by older participants, men, and non-healthcare workers than their counterparts. Thus, the motivations of those choosing to participate and respond may differ from that of the general population. The specific questions asked were not identical to the CDC guidelines; for example, CDC recommended consideration of SARS-CoV-2 testing only prior to travel, not routinely before attending a gathering [8]. The size of the gatherings and the exact number of NHM at those gatherings were not quantified. The two surveys encompassed holidays of differing lengths of time (4-days for Thanksgiving vs. over 2-weeks for the winter holidays). Lastly, the survey did not inquire about plans for post-holiday COVID-19 testing or whether those participating in gatherings with NHM intended to quarantine prior to travel or for 14 days afterward.

In conclusion, despite warnings from public health authorities that Thanksgiving and WH season travel and participation in gatherings with NHM might exacerbate the evolving surge in COVID-19 cases [2, 4, 8], about half of respondents chose to travel and pursue traditional gatherings. In addition, only ~ 40% of study participants (37.3% at Thanksgiving and 41.9% during the winter holidays) reported wearing a mask or face covering when gathering with others outside their household. These data raise questions about whether inadequate adoption of recommended safe COVID-19 behaviors over the holidays may have contributed to the well-documented post-holiday surge in COVID-19 cases, and highlight the need for more effective ways to promote all recommended safe COVID-19 behaviors in the future. In conjunction with vaccination, the ongoing selective utilization of preventive behaviors may be important to ensure that increases in COVID-19 infections do not occur as pre-pandemic activities are resumed [21].

## Data Availability

The datasets used and/or analyzed during the current study are available from the corresponding author on reasonable request.
